# Systematic Map of Human–Raptor Interaction and Coexistence Research

**DOI:** 10.3390/ani12010045

**Published:** 2021-12-27

**Authors:** Angeline C. Canney, Lauren M. McGough, Nate A. Bickford, Kenneth E. Wallen

**Affiliations:** 1 Department of Biology, Colorado State University Pueblo, Pueblo, CO 81001, USA; angcanney@gmail.com (A.C.C.); lauren.mcgough@csupueblo.edu (L.M.M.); nate.bickford@csupueblo.edu (N.A.B.); 2 Department of Natural Resources and Society, University of Idaho, Moscow, ID 83844, USA

**Keywords:** conservation social sciences, human dimensions, human–wildlife conflict, illegal shooting, persecution

## Abstract

**Simple Summary:**

Raptors are affected by interactions with humans, primarily in the form of persecution and habitat disturbance. Here, we quantify and characterize empirical literature on human–raptor interactions, inclusive of sociocultural, ecological, natural history, and conservation perspectives. We focus on species, geography, and human-caused mortality to understand the scope of research and directions for future raptor conservation research. Although raptor conservation is intrinsically the study of human behavior and social systems, we found ecological research that focuses on the effects humans have on raptors encompasses the majority of human–raptor interaction research. We stress the need to focus on the causes of human–raptor interactions and suggest that the combination of social, ecological, and management-relevant approaches is best to examine problems and identify solutions.

**Abstract:**

Global raptor conservation relies on humans to establish and improve interaction and coexistence. Human–wildlife interaction research is well-established, but tends to focus on large-bodied, terrestrial mammals. The scope and characteristics of research that explores human–raptor interactions are relatively unknown. As an initial step toward quantifying and characterizing the state of applied, cross-disciplinary literature on human–raptor interactions, we use established systematic map (scoping reviews) protocols to catalog literature and describe trends, identify gaps and biases, and critically reflect on the scope of research. We focus on the peer-reviewed (refereed) literature germane to human–raptor interaction, conflict, tolerance, acceptance, persecution and coexistence. Based on 383 papers retrieved that fit our criteria, we identified trends, biases, and gaps. These include a majority of research taking place within North America and Europe; disproportionately few interdisciplinary and social research studies; interactions focused on indirect anthropogenic mortality; and vague calls for human behavior changes, with few concrete steps suggested, when management objectives are discussed. Overall, we note a predominant focus on the study of ecological effects from human–raptor interactions rather than sociocultural causes, and suggest (as others have in various conservation contexts) the imperative of human behavioral, cultural, and political inquiry to conserve raptor species.

## 1. Introduction

Raptors are negatively affected by interactions with humans, primarily in the form of anthropogenic disturbances and persecution [1,2]. Environmental contaminants and habitat loss are persistent threats, but direct (intentional) and indirect (unintentional) mortality from a constellation of human behaviors—shooting, trapping, poisoning, and poaching—and human-made structures—wind turbines, buildings, and powerlines—are likewise constant. Recognition that human actions are the cause of conservation challenges but also fundamental to the solutions is imperative [3,4]. To conserve raptors, it is essential that the human dimensions related to persecution, conflict, coexistence, tolerance, and acceptance are recognized and addressed.

In this respect, the field of human–wildlife interaction (also referred to as human–wildlife conflict) and coexistence is key [5]. Interaction with wildlife is a ubiquitous human experience; humans compete with wildlife for material resources, attach to them sociocultural or spiritual value, and embed intergenerational positive to negative connotations associated with those interactions [6,7]. However, research on human–wildlife interaction and coexistence tends to focus on large terrestrial mammals such as elephants, bears, felids, and canids [8].

Empirical research on human–raptor interactions has been relatively limited to efforts that explore and describe broad-scale ecological factors that affect populations [9,10]. Some speculate that human actions such as shooting or trapping, which are viewed as affecting individuals rather than populations, are of little ecological consequence compared to large scale issues of habitat and prey loss [6]. Recent evidence suggests that this claim is tenuous. Among some species, e.g., golden eagle (*Aquila chrysaetos*), shooting is now recognized as the leading form of mortality [2]. Coupled with the cumulative effects of climate change, habitat loss, and issues that affect prey species, the role of anthropogenic mortality and human behavior can no longer be ignored. In recent years, research that applies theories and methods designed to observe, measure, explain and generalize anthropogenic mortality and human behavior has become more common [11,12,13].

Given the various perspectives that now explore human–raptor interactions and coexistence, it is essential to systematically collate, catalog, describe, and assess the state of published research. Knowledge of the extant body of cross-disciplinary literature is necessary to advance our understanding of the causes and consequences of negative human–raptor interactions and avenues for coexistence. To that end, our objective is to use established guidelines to produce a systematic map of existing empirical, peer-reviewed human–raptor interaction and coexistence research. Our intent is to advance the human dimensions of raptor conservation via the identification of research trends and gaps, characterization of mainstream features of the literature, and description of the scope of research in the field. From our perspective, advancing raptor conservation in the Anthropocene requires that the human dimensions are made focal to facilitate novel questions, improve actionability, and reflect on past, present, and future research and practice.

Our objective is to quantify and characterize the empirical, peer-reviewed literature that measures an aspect of anthropogenic interaction with raptors, inclusive of sociocultural, ecological, natural history, and conservation perspectives. Research on human–raptor interactions is cross-disciplinary and draws on ecological, political, archival, economic, and social research, as well as the work of conservation, law, enforcement, and rehabilitation practitioners. Our effort is inclusive of disciplines and domains that attempt to address the human dimensions of raptor conservation, e.g., persecution, interaction, conflict, coexistence, tolerance, and acceptance. We include a breadth of empirical biological and sociocultural raptor research on indirect and direct anthropogenic mortalities, anthropogenic effects on populations and conservation status, human belief systems, and resultant behaviors towards or affecting raptors. Our effort is guided by the following research questions: (a) what is the scope of published research, (b) what research contexts are represented, (c) what research designs, methods, and sampling are used, (d) what human factors and mortality types are focal and commonly measured, (e) what mitigation strategies are tested or recommended, and (f) what can be inferred from this body of empirical, peer-reviewed research to guide future research?

## 2. Materials and Methods

Systematic maps are an important tool to aggregate and describe a body of literature across a specific topic [14]. Systematic maps are appropriate to assess how much research is available on a topic within the scope of explicit questions and with the intention to identify and assess gaps and trends [15]. In general, the objective is to understand what research has been conducted, i.e., where, how, and in what form [16,17]. The resultant catalog of the literature that is created via a systematic map protocol establishes a searchable database that allows researchers to review, use, and update as well as identify areas with sufficient representation to allow for a systematic review.

Our methodology followed guidelines established by the Collaboration for Environmental Evidence [18]. We complied with both PRISMA and ROSES reporting standards [15,17,19]. Our methodological choices ensure a transparent and standardized design that is repeatable and comparable [20]. See Supplementary Materials for reporting standard checklists.

Our search strategy consisted of four stages: identification, screening, eligibility, and inclusion. Identification comprised a systematic literature search of peer-reviewed journals using two established research databases: Web of Science Core Collection (via the Web of Science platform) and Academic Search Premier (via the EBSCOhost research platform). Searches were conducted in April 2021. The search did not set date parameters, so all possible records within each database up to the search date could be retrieved.

We developed an English-language search string to collect records relevant to our inquiry. The parameters of the search strategy were developed iteratively and employed both external expert review and benchmark article protocols. The search string was: (“raptor *” or “bird * of prey” or “vulture *” or “condor *” or “eagle *” or “owl *” or “buzzard *” or “hawk *” or “falcon *” or “harrier *” or “kite *” or “osprey *” or “secretary bird *” or “kestrel” or “merlin” or “gyrfalcon”) and (“persecution *” or “conflict *” or “coexistence” or “tolerance” or “acceptance” or “human-relat *” or “shoot *” or “poison *” or “poach *” or “lethal control” or “illegal kill *” or “illegal hunt *” or “vermin”) not (“raptorial” or “falconeri”). The parent search string was augmented to accommodate the parameters of each database.

Screening began with first removing duplicate entries based on the digital object identifier (DOI) and title. Four coders (authors AC, LM, NB, KW) then manually screened records by title and abstract (full text, as needed) to judge correspondence with eligibility criteria and eliminate irrelevant records. The eligibility stage focused on our predetermined inclusion criteria that each publication (1) reported or reviewed empirical research, (2) focused on human cognition, human behavior, or societal activities in the context of raptors or the interaction between raptors and human behavior and/or social process, and (3) measured some aspect (variable) of human cognition, human behavior, or societal activity. Intercoder reliability issues associated with consistency and agreement were resolved via consensus.

The inclusion stage consisted of full-text screening. The four coders were randomly assigned an equal number of the retrieved records to independently code (*n* = 377). Records relevant to the scope of inquiry were coded for the following attributes: study location (country), study species (group), research focus (e.g., biological, social), study context (e.g., conservation, conflict, natural history, sociocultural), research design, methodology, study population (human), sampling design, human–raptor interaction framing (e.g., conflict, coexistence), measured human factors (e.g., behavior, attitude), intentional action, mortality type, and mitigation strategy. Standard bibliometric attributes were appended to the codebook: title, author(s), source, publication year, keyword, abstract, and DOI.

A narrative synthesis approach was applied to identify trends and gaps, characterize mainstream features of the literature, and describe the scope of research in the field through the use of descriptive statistics, tables, and figures [21]. Narrative synthesis uses a textual approach to synthesize and tell the story of the findings based on the studies included. All data analyses were conducted in R, and descriptive analyses and bar charts were produced using base R or ggplot2.

## 3. Results

**Figure 1:**
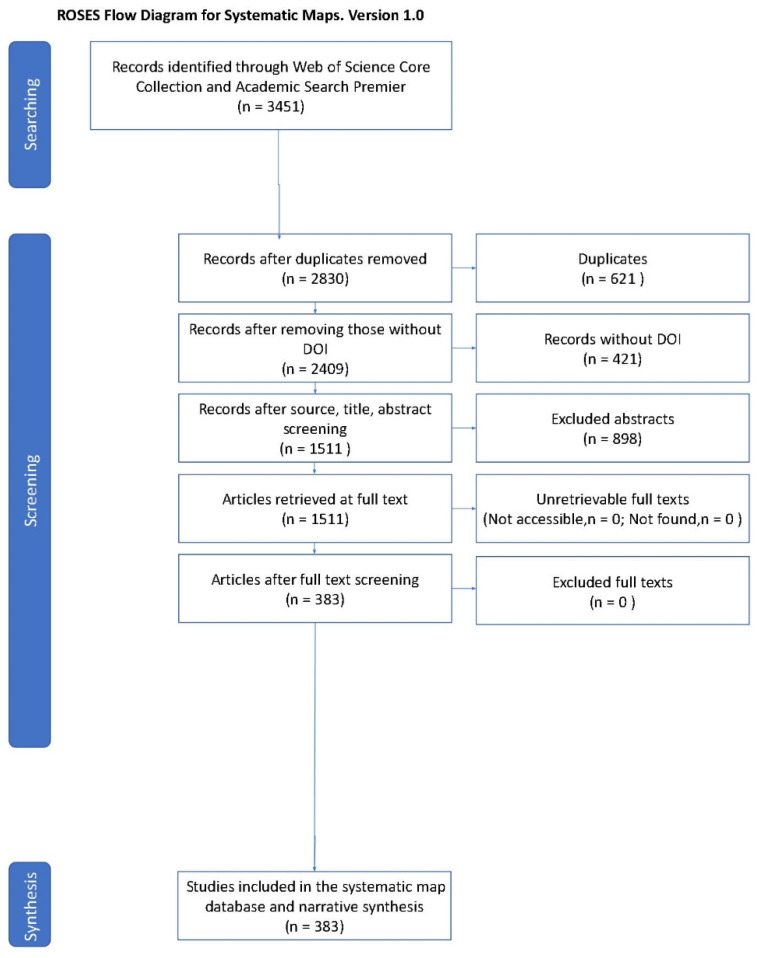
Search and review ROSES flow diagram for a systematic map of human–raptor interaction and coexistence research.

The systematic search of the literature yielded *n* = 3451 records. The number of records after duplicates were eliminated was reduced to *n* = 2830 unique records. Title, records without a DOI, and abstract screening reduced the number of potential records to 2409, and full-text screening yielded 383 articles for analysis (Figure 1). A qualitative intercoder reliability process was used to assess the consistency of inclusion and exclusion, wherein 10% (*n* = 38) of records were independently coded by all four authors; all discrepancies were discussed to reach a consensus.

Species from *Accipitridae* (62.4%) were the most represented group among the records retrieved. We used the code “Multiple families” to indicate papers that focused on three or more different species of raptorial birds (17.2%). A similar number of studies were found to focus on *Strigidae* (*n* = 26), *Cathartidae* (*n* = 22), and *Falconidae* (*n* = 20) families (Table 1) (please note, for all results, coded categories were not necessarily mutually exclusive, and therefore counts may not all sum to *n* = 383).

To understand the social and ecological focus of human–raptor interactions, we categorized publications into three general types: “human factor focus” (*n* = 60, 15.9%), “ecological factor focus” (*n* = 308, 81.6%), or a “non-specific focus” (*n* = 9, 2.3%) (Table 2). Human factor publications were defined as studies that focused on measuring human behavior (manifest) or cognition (latent); for example, measuring the traditional beliefs of community members in relation to vulture poaching [22]. Ecological publications were defined as studies that generally focused on measuring the effects of human actions on individual raptors, raptor populations, and habitat; for example, the impact of wind farms on raptor species [23].

We coded context to represent the general orientation or focus of inquiry implied or explicitly stated in a paper. “Human–wildlife conflict” (*n* = 250) was most frequent, followed by “conservation” (*n* = 168), “life/natural history” (*n* = 134), and “sociocultural” (*n* = 38). We coded framing to further examine the semantic or rhetorical framework authors used to represent or describe the human–raptor relationship [8]. “Conflict” (*n*= 105) was the most frequently used descriptive, followed by “interaction” (*n* = 80), “coexistence” (*n* = 74), “persecution” (*n* = 30), “tolerance” (*n* = 16), and “acceptance” (*n* = 7).

Human factors were coded to examine the conceptual (theoretical) or operational (measurement) variables that were focal. Human factors included “attitude” (*n* = 93), “value” (*n* = 63), “norm”, (*n* = 47), “belief” (*n* = 45), “intentions” (*n* = 38), “awareness” (*n* = 33), “knowledge” (*n* = 30), “motivation” (*n* = 23), “identity” (*n* = 22), “attachment” (*n* = 16), “intentions (*n*= 14), “experience” (*n* = 9), “morals” (*n* = 5), “emotions” (*n* = 4), “violence” (*n* = 3), “risk”, (*n* = 2), and “policy/regulation” (*n* = 2).

The highest mortality coded was multiple mortalities (*n* = 75, 15.2%), which represents papers that identified more than two mortalities (Table 3). “Lead” was the second-highest identified mortality (*n* = 71, 14.5%). “Other poison” was further specified into “pesticide” (*n* = 26, 5.3%) and “rodenticide” (*n* = 19, 3.8%), when possible. Further separation within mortality was identified as “unintentional” (*n* = 152, 40.3%) or “intentional” (*n* = 52, 13.8%). Overall Accipitridae was the most studied raptor group for mortality (*n* = 243), and the most frequent mortality was “other poison” (*n* = 45).

We coded mitigation to examine the suggestions and considerations that the authors included (primarily in the discussion sections of papers) to mitigate human–raptor interactions. Some papers were coded for multiple mitigations, and “human behavior change” (*n* = 103, 21.4%) was the most commonly mentioned mitigation strategy. This was followed by “policy/regulation changes” (*n* = 90, 18.7%); however, most studies did not suggest mitigation practices—“none” (*n* = 154, 32%).

**Figure 2:**
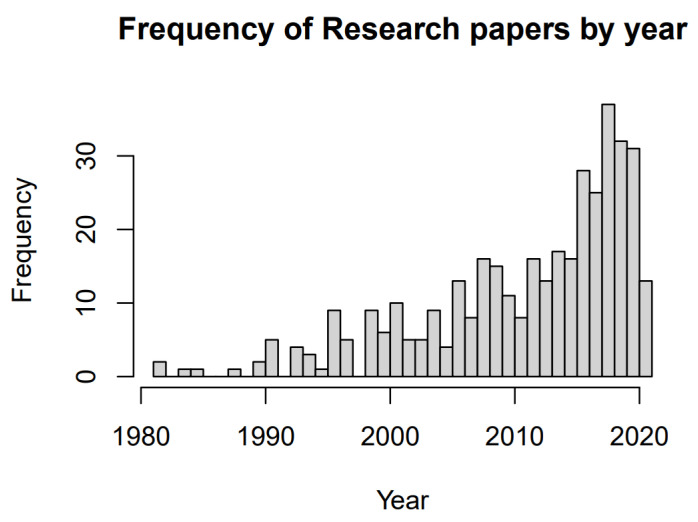
Frequency of publications by year a systematic map of human–raptor interaction and coexistence research. Please note that the search was conducted in April 2021, and therefore 2021 is not fully represented in the results.

In terms of bibliometrics, 120 journals were represented in the final analysis. Of those, 21 journals had at least five publications, which represented 55.2% of all publications. Since the 1980s, there has been an upward trend in publications: 1980s—*n* = 5, 0.5 articles/year; 1990s—*n* = 38, 3.8 articles/year; 2000s—*n* = 92, 9.2 articles/year; 2010s—*n* = 203, 20.3 articles/year; and 2020–21—*n* = 44, 22 articles/year (Figure 2). *Biological Conservation* and *Journal of Raptor Research* (*n* = 35) had the most publications, both in terms of socially and ecologically focused human–raptor interactions.

**Figure 3:**
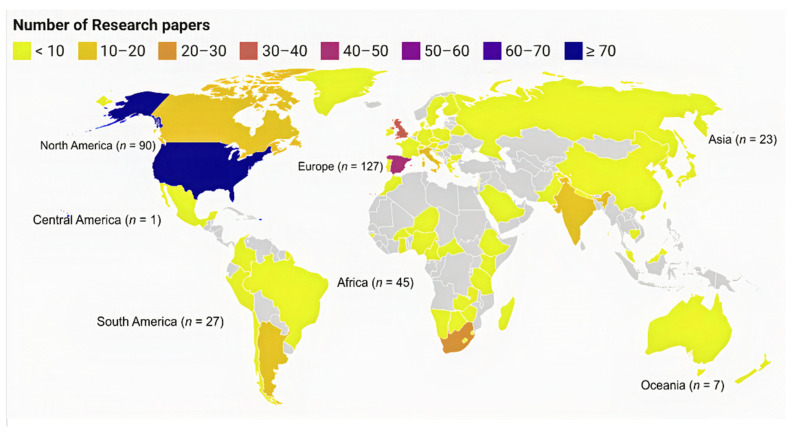
Distribution of the reported study location (country) based on 383 publications identified in a systematic map of human–raptor interaction and coexistence research.

In terms of geography, 68 countries were represented (Figure 3). All continents were represented (excluding Antarctica), with research papers distributed in the following regions: North America (*n* = 90), Europe (*n* = 127), Asia (*n* = 23), South America (*n* = 27), Central America (*n* = 1), Oceania (*n* = 7), Africa (*n* = 45), and worldwide (*n* = 5). The United States (*n* = 73) and Spain (*n* = 47) were observed to have the most studies, while Great Britain (*n* = 8) represented the country with the most socially focused studies.

In terms of research design, approximately half were coded as a descriptive studies (51.8%), and one-fifth as case studies (20%). Please note, design categories were not mutually exclusive, i.e., a study can have multiple designs. Method was defined as how data were collected. “Observational” (*n* = 139) studies were most common, followed by “archival, historical” (*n* = 85), “computational” (*n* = 73), “ necropsy” (*n* = 71), “synthesis” (*n* = 59), “quantitative survey” (*n* = 37), “mixed methods” (*n* = 37), “secondary/specimen survey” (*n* = 35), “spatial” (*n* = 34), “qualitative survey”, (*n* = 24), “participatory” (*n* = 12), and “band recovery” (*n* = 2). In terms of sampling protocols used by socially focused studies, non-random sampling protocols were most common (25.7%) (Table 4). We also examined the study population, the most prevalent being “farmer” (*n* = 21) and “resident” (*n* = 19).

## 4. Discussion

The human dimensions are imperative to raptor conservation and coexistence. Our systematic assessment of the human–raptor interaction literature—to identify trends, gaps, mainstream features, and the scope of research—reveals a field that has continually grown since the early 1980s. Our main findings and contributions to raptor conservation are empirical observations that the majority of human–raptor interaction research (a) is conducted in Europe (Spain and Great Britain) and North America (United States), (b) focuses on species in the *Accipitridae* family, (c) studies indirect and unintentional forms of mortality (poisoning and collisions with human infrastructure) more commonly than direct and intentional forms (shooting and trapping), and (d) focuses on the ecological effects of negative human–raptor interactions rather than the sociocultural causes of these ecological effects. In addition, the majority of the mitigation strategies suggested were related to human behavior change and policy/regulation, even though this research focus was underrepresented compared to ecological research.

The fact that the vast majority of research reviewed took place in North America and Europe is not unexpected. Although our review was limited to English-language publications (which is common among scoping reviews and systematic maps), previous structured reviews and systematic maps of conservation issues reveal a similar bias [24,25,26,27]. Areas with diverse, important, and declining populations of raptors such as Africa, Asia, and South America were underrepresented in the literature and, in particular, raptor families and species endemic to these areas [1]. As an example, it was particularly striking to know that Indonesia contains both the highest raptor species richness and highest rates of decline and observe no papers in this review. It must be said that our approach to the systematic map procedure does have its limitations and is prone to biases associated with search terms and language, and there may, in fact, be past or present research on human–raptor interactions in Indonesia. This example highlights the need for more concerted and, perhaps, collaborative effort to focus on regions with little or no ongoing research beyond the current infrastructure and capabilities [28].

Issues of obtaining research permits, finding collaborators, the presence of armed conflicts, and the underfunding of science are practical and logistical reasons that could explain these trends. However, there remains the possibility that research conducted in developing countries is unlikely to be published in leading journals, resulting in a geographic biases [26], or that center-periphery dynamics shift researchers away from the Global South [24]. The Global North tends to have the research capacity and financial/institutional support to address applied conservation problems, such that established funding–research–publication pathways exist and are readily accessible. Many countries and regions have no published research or less than a handful of published human–raptor interaction research papers. We contend that this is a tenuous and unsustainable pattern that has been and continues to be a contributory cause of declining raptor populations globally. As we reiterate below, conservation is a human behavior with social, cultural, or political causes that must be empirically examined and understood [4].

*Accipitridae* are the focal species in nearly two-thirds of the records reviewed. While this family does constitute a large proportion of all raptor species, we observed a disproportional decline in relation to research on *Strigidae*, *Cathartidae*, and *Falconidae*. Based on the findings, there seems to be a large gap in our knowledge of human–raptor interaction among many species, both common and threatened. How a species gets chosen as the focus of a research project varies. However, there is a balance to be struck between research on charismatic species, the species expertise of a researcher, or, for example, the guiding principle “keep common species common”, and the priority that must be given to threatened and endangered species. For example, basic research to understand the dynamics of either the ecological or sociocultural dimensions of human–raptor interaction must eventually transform to applied and actionable research on species of conservation concern. This requires transdisciplinary and team science approaches to the co-development and co-design of human–raptor interaction research [29,30,31].

Our findings show that the most of anthropogenic raptor mortalities studied were unintentional (indirect) mortalities, with lead toxicosis and wind turbine collisions being prominently represented in the literature (the latter being likely correlated with the North American research bias observed). As human activities and development have and continue to encroach on raptor habitat and the habitat of prey species, we do not see the issue lessening without proactive mitigation. In North America, mitigation and partnerships between private and public institutions has led to the decline of raptor electrocution via utility line retrofits [32]. The findings also highlight the prevalence of poisoning, rodenticides, pesticides, and lead, among others, as a direct and indirect mortality type. This includes direct poisoning that aims to intentionally mass kill raptors, e.g., vultures killed by poachers, and indirect poisoning as a consequence of other human activity, e.g., the veterinary drug diclofenac.

Direct mortality in the form of shooting and trapping comprised a disproportionately low number of records compared to updated estimates of mortality type among raptors [2]. Compounding this issue is the disproportionate focus on ecological effects rather than sociocultural causes, with a dearth of the research literature dedicated to examining the motivations of intentional killing, e.g., food, sport, traditional belief-based use, and the perception that raptors damage game populations or livestock. Considering that very well-studied and legally protected populations such as golden eagles in North America and hen harriers in the United Kingdom suffer from significant direct mortality, it is likely indicative of a larger problem [33]. We surmise that direct anthropogenic mortality, particularly in the form of shooting, trapping, and poisoning, is likely an underreported source of mortality among many raptor species. Rightly, the human dimensions are the most difficult and complex component of raptor conservation and natural history, particularly as (a) killing raptor species is an illegal behavior in North American and European countries and (b) many culturally embedded belief systems encompass raptors specifically and predator species in general.

The lack of research focused on the behavioral, cultural, and political dimensions of human–raptor interactions was quite prominent; a focus on these phenomena was absent from nearly 80% of the papers reviewed. It has been astutely said that “conservation is a human endeavor: initiated by humans, designed by humans, and intended to modify human behavior” [34]. Of the papers analyzed, less than one-fifth directly measured individual human behaviors towards or beliefs about raptors, while the majority measured the effect human actions had on raptor populations. Again, the human–raptor interaction literature is heavily weighted towards empirical research of effects rather than causes. A minority of papers attempted to understand the underlying social, cultural, or political causes and motivations for negative human–raptor interaction that likely drive conflict and conservation issues in many areas.

We resolutely bring attention to this major gap in the human–raptor interaction literature. Where human populations were studied to better understand behavior and belief and value systems, residents and farmers (which we acknowledge are not the most descriptive categories) were the populations most frequently studied. We found this focus to be logical, since a focus on residents in an area with prevalent raptor persecution is important to understanding that persecution, and agriculture is the most common threat identified for global raptor populations [35,36]. However, we note an overfocus on general and broadly defined behavioral or sociocultural concepts such as attitudes and values—which is also a trend among general human dimensions of conservation research [37]—as opposed to other concepts that may be closer antecedents to behavior, such as experience, intentions, mortality, risk perception, and emotions. As the human dimensions of raptor conservation grow and presumably increase its focus on human–raptor interactions, we would urge researchers and practitioners to consider the spectrum of internal and external influences on human behavior [38].

Interestingly, while the majority of papers focused on the ecological effects of human–raptor interaction, the suggested mitigation measures for these effects included aspects of human dimensions such as behavior change and policy/regulation. However, rarely were the steps, means, and research needed to achieve such changes ever specified, which is likely a side-effect of inadvertently studying effects without also studying causes. One can assume various reasons for what we perceive as cursory mitigation statements and suggestions. In our experience, these statements in discussion or conclusion sections, while seemingly earnest (we ourselves have made such statements), are also possibly remnants of the peer review process (i.e., to appease reviewers), attempts to interject management implications to research that was not specifically designed with managers to inform management (or similarly not designed with social researchers to understand social elements), or recognition by ecological-focused researchers that the sociocultural is the imperative to understanding and solving human–raptor interaction issues. As stated above, remediation of this issue requires researchers to make opportunities for the interaction and integration of multiple perspectives and disciplines, specifically those researchers and approaches that focus on the social, cultural, or political methodologies to observe, measure, and explain human cognitive and behavioral phenomena.

## 5. Conclusions

The central contribution of this systematic map of human–raptor interactions is the quantification and characterization of empirical, peer-reviewed (referred) literature that measures human–raptor interactions, inclusive of sociocultural, ecological, natural history, and conservation perspectives. Beyond the empirical findings, our efforts make clear the need to integrate social, ecological, and management-relevant approaches to address the challenges of and identify solutions raptor conservation. From our perspective, raptor conservation is intrinsically the study of human behavior and social processes. An integral step towards more human–raptor interaction literature that informs conservation, and, indeed, effective raptor conservation is the establishment of transdisciplinary and team-based research collaborations that integrate and prioritize the human dimensions. Raptor populations are sensitive to a wide scope of anthropogenic change and mortality, many of which (such as collisions with infrastructure and wind turbines) are only growing, and other direct forms (such as shooting, trapping and poisoning) have not been quantified in areas with diverse and declining raptor populations, representing gaps in our knowledge of anthropogenic mortality. Advancing raptor conservation in the Anthropocene requires that the human dimensions are made focal so as to facilitate novel questions, improve actionability, and to reflect on past, present, and future research and practice. To truly make positive impacts on many of these raptor populations, we need to understand the human element.

## Figures and Tables

**Table 1:** Raptor family frequency within the reviewed human-raptor interaction research.

Family	Frequency (%)
*Accipitridae*	243 (62.4)
*Multiple families*	67 (17.2)
*Strigidae*	27 (6.9)
*Cathartidae*	23 (5.9)
*Falconidae*	23 (5.9)
*Pandionidae*	4 (1)
*Otididae*	1 (<1)
*Unknown*	1 (<1)

**Table 2:** Frequency of study focus and context within the reviewed human-raptor interaction research.

Variable	Number of Articles (%)
**Study Focus**	
Ecological	308 (81.6)
Human	60 (15.9)
Non-Specific	9 (2.3)
**Study Context**	
Human–wildlife conflict	250 (42.4)
Conservation	168 (28.5)
Life/Natural history	134 (22.7)
Sociocultural	38 (6.4)

**Table 3:** Mortality types and frequencies documented within the reviewed human-raptor interaction research.

Mortality	Frequency (%)
Multiple mortalities	75 (15.2)
Lead	71 (14.5)
Other poison	70 (14.2)
Collision (wind)	58 (11.8)
Shooting	41 (8.3)
Pesticide	26 (5.3)
Rodenticide	19 (3.8)
Electrocution	11 (2.2)
Non-specific/trauma	10 (2.0)
Collision (auto)	9 (1.8)
Trapping	8 (1.6)
Collision (structure)	8 (1.6)
Development	6 (1.2)

**Table 4:** Methodology used within the reviewed human-raptor interaction research.

Methodology	Frequency (%)
Observational	139 (22.9)
Archival, Historical	85 (14.0)
Computational	73 (12.0)
Necropsy	71 (11.7)
Synthesis	59 (9.7)
Mixed method	37 (6.1)
Quantitative survey	37 (6.1)
Secondary/Specimen survey	35 (5.8)
Spatial	34 (5.6)
Qualitative survey	24 (3.9)
Participatory	12 (2.0)
Band Recovery	2 (0.3)

## Data Availability

Data, Supplementary Materials, and documentation are available online via the following link: https://figshare.com/projects/Systematic_map_of_human–raptor_interaction_and_coexistence_research/127547 (accessed on 20 December 2021).

## Supplementary Materials

The following are available online at https://www.mdpi.com/article/10.3390/ani12010045/s1. Supplementary file: Human-raptor interaction systematic map supplementary information.
