# Supplementary material for: Systematic Map of Human–Raptor Interaction and Coexistence Research
Source: Animals (Basel). 2021 Dec 27;12(1):45. doi: 10.3390/ani12010045 (PMC8749526; doi:10.3390/ani12010045)
Supplement: Supplementary file 1 [file animals-12-00045-s001.zip › animals-1514241-supplementary materials.pdf]

Canney AC, McGough LM, Bickford NA, Wallen KE. 2021. Systematic map of human-raptor interaction and coexistence research.

**ROSES form for systematic map report.**

https://doi.org/10.6084/m9.figshare.17108666

Canney AC, McGough LM, Bickford NA, Wallen KE. 2021. Systematic map of human-raptor interaction and coexistence research.

**Literature search string.**

("raptor*" OR "bird* of prey" OR "vulture*" OR "condor*" OR "eagle*" OR "owl*" OR "buzzard*" OR "hawk*" OR "falcon*" OR "harrier*" OR "kite*" OR "osprey*" OR "secretary bird*" OR “kestrel” OR “merlin” OR “gyrfalcon”) AND ("persecution*" OR "conflict*" OR "coexistence" OR "tolerance" OR "acceptance" OR “human-relat*” OR “shoot*” OR “poison*” OR "poach*" OR “lethal control” OR "illegal kill*" OR "illegal hunt*" OR “vermin”) NOT ("raptorial" OR "falconeri").

Canney AC, McGough LM, Bickford NA, Wallen KE. 2021. Systematic map of human-raptor interaction and coexistence research.

**Benchmark list.**


Almuna, R., Cruz, J. M., Vargas, F. H., & Ibarra, J. T. (2020). Landscapes of coexistence: Generating predictive risk models to mitigate human-raptor conflicts in forest socio-ecosystems. Biological Conservation, 251, 108795. https://doi.org/10.1016/j.biocon.2020.108795

Hager, S. B. (2009). Human-Related Threats to Urban Raptors. Journal of Raptor Research, 43(3), 210–226. https://doi.org/10.3356/JRR-08-63.1

Hunt, W. G., Wiens, J. D., Law, P. R., Fuller, M. R., Hunt, T. L., Driscoll, D. E., & Jackman, R. E. (2017). Quantifying the demographic cost of human-related mortality to a raptor population. PLOS ONE, 12(2), e0172232. https://doi.org/10.1371/journal.pone.0172232

Kumar, N., Jhala, Y. V., Qureshi, Q., Gosler, A. G., & Sergio, F. (2019). Human-attacks by an urban raptor are tied to human subsidies and religious practices. Scientific Reports, 9(1), 2545. https://doi.org/10.1038/s41598-019-38662-z

Kumar, N., Qureshi, Q., Jhala, Y. V., Gosler, A. G., & Sergio, F. (2018). Offspring defense by an urban raptor responds to human subsidies and ritual animal-feeding practices. PLOS ONE, 13(10), e0204549. https://doi.org/10.1371/journal.pone.0204549

Madden, K. K., Rozhon, G. C., & Dwyer, J. F. (2019). Conservation Letter: Raptor Persecution. Journal of Raptor Research, 53(2), 230–233. https://doi.org/10.3356/JRR-18-37

Redpath, S. M., Arroyo, B. E., Leckie, F. M., Bacon, P., Bayfield, N., Gutiérrez, R. J., & Thirgood, S. J. (2004). Using Decision Modeling with Stakeholders to Reduce Human–Wildlife Conflict: A Raptor–Grouse Case Study. Conservation Biology, 18(2), 350–359. https://doi.org/10.1111/j.1523-1739.2004.00421.x

Restrepo-Cardona, J. S., Echeverry-Galvis, M. Á., Maya, D. L., Vargas, F. H., Tapasco, O., & Renjifo, L. M. (2020). Human-raptor conflict in rural settlements of Colombia. PLOS ONE, 15(1), e0227704. https://doi.org/10.1371/journal.pone.0227704

Rodríguez, B., Rodríguez, A., Siverio, F., & Siverio, M. (2010). Causes of Raptor Admissions to a Wildlife Rehabilitation Center in Tenerife (Canary Islands). Journal of Raptor Research, 44(1), 30–39. https://doi.org/10.3356/JRR-09-40.1

Tavecchia, G., Adrover, J., Navarro, A. M., & Pradel, R. (2012). Modelling mortality causes in longitudinal data in the presence of tag loss: Application to raptor poisoning and electrocution. Journal of Applied Ecology, 49(1), 297–305. https://doi.org/10.1111/j.1365-2664.2011.02074.x

Thirgood, S., & Redpath, S. (2008). Hen harriers and red grouse: Science, politics and human–wildlife conflict. Journal of Applied Ecology, 45(5), 1550–1554. https://doi.org/10.1111/j.1365-2664.2008.01519.x

Canney AC, McGough LM, Bickford NA, Wallen KE. 2021. Systematic map of human-raptor interaction and coexistence research.

**Codebook and Systematic map database.**

https://doi.org/10.6084/m9.figshare.17108651
